# Could Extracellular Vesicles Contribute to Generation or Awakening of “Sleepy” Metastatic Niches?

**DOI:** 10.3389/fcell.2021.625221

**Published:** 2021-03-02

**Authors:** Alberto Hernández-Barranco, Laura Nogués, Héctor Peinado

**Affiliations:** Microenvironment and Metastasis Laboratory, Molecular Oncology Programme, Spanish National Cancer Research Center (CNIO), Madrid, Spain

**Keywords:** extracellular vesicle, exosome, dormancy, metastasis, disseminated tumor cells

## Abstract

Pre-metastatic niches provide favorable conditions for tumor cells to disseminate, home to and grow in otherwise unfamiliar and distal microenvironments. Tumor-derived extracellular vesicles are now recognized as carriers of key messengers secreted by primary tumors, signals that induce the formation of pre-metastatic niches. Recent evidence suggests that tumor cells can disseminate from the very earliest stages of primary tumor development. However, once they reach distal sites, tumor cells can persist in a dormant state for long periods of time until their growth is reactivated and they produce metastatic lesions. In this new scenario, the question arises as to whether extracellular vesicles could influence the formation of these metastatic niches with dormant tumor cells? (here defined as “sleepy niches”). If so, what are the molecular mechanisms involved? In this perspective-review article, we discuss the possible influence of extracellular vesicles in early metastatic dissemination and whether they might play a role in tumor cell dormancy. In addition, we comment whether extracellular vesicle-mediated signals may be involved in tumor cell awakening, considering the possibility that extracellular vesicles might serve as biomarkers to detect early metastasis and/or minimal residual disease (MRD) monitoring.

## Introduction

There is evidence suggesting that tumor cells disseminate from the very beginning of primary tumor formation (Hosseini et al., 2016). Disseminated tumor cells (DTCs) in circulation eventually reach specific distal sites where these metastatic cells become quiescent (Goddard et al., 2018). This phenomenon, known as tumor dormancy, could be maintained for several years before these cells reactivate to generate secondary lesions, explaining how metastasis can appear in cancer patients with no evidence of disease after successful treatments, even after complete resection of the primary tumors. The knowledge that dissemination happens early in tumor development has challenged traditional models of metastatic progression, representing a change in the paradigm that metastatic cells only appear late on in tumor progression (Harper et al., 2016; Suhail et al., 2019). Regardless of the mechanisms involved, metastasis is considered an inefficient process (Luzzi et al., 1998) as most DTCs that leave the primary tumor die through apoptosis or immune clearance along their journey (Mehlen and Puisieux, 2006). As such, only a few of them successfully reach distal organs, extravasate and once there, survive in a quiescent state. The specific mechanisms underlying tumor latency in dormant niches is beginning to be defined, suggesting a key role for the microenvironment in these niches (Bissell and Hines, 2011). The balance between DTC dormancy and cell awakening is conditioned by signals either from tumor cells or from stromal components within the surrounding area, including signals from the extracellular matrix (ECM), the vasculature and the immune system (Ghajar, 2015; Aguirre-Ghiso, 2018). Evidence has accumulated over recent years that the niche surrounding the microvasculature (e.g., the perivascular niche -PVN) orchestrates DTC dormancy, principally responsible for cell survival and growth arrest (Ghajar, 2015). There is also strong evidence that certain ECM proteins promote cell dormancy, such as the thrombospondin-1 (THBS1) of the microvascular endothelium in breast cancer (Ghajar et al., 2013) and osteopontin within the bone marrow in leukemia (Boyerinas et al., 2013). Some ECM factors like fibronectin induce entry into a dormant cell phenotype, which is dependent on soluble factors like transforming growth factor-β (TGFβ), Barney et al., 2020) suggesting that ECM and soluble factors may join forces to regulate DTC dormancy. However, other works also showed that fibronectin reawakes dormant tumor cells (Barkan et al., 2008; Eyles et al., 2010), supporting that depending on the model used, fibronectin may have a differential role regulating dormancy/awakening.

Interestingly, extracellular vesicles (EVs) have emerged as important messengers in cell-cell communication (Tkach and Thery, 2016), although the contribution of EVs to tumor cell dormancy is still poorly understood. As such, we will discuss here the potential role of EVs in the communication between tumor and stromal cells, and their influence on tumor cell dormancy and awakening in metastasis.

## Microenvironmental Regulation of DTC Dormancy

Metastatic lesions preferentially develop in specific anatomical locations (Paget, 1989), suggesting that a combination of intrinsic and extrinsic factors dictates the success of DTC colonization (Bragado et al., 2012). The characteristics of the dormant niche include the changes to the microenvironment that favor metastatic cell survival (e.g., stem cell properties, immune, and endothelial cell changes) (Goddard et al., 2018), which together with the ECM and hypoxic microenvironments (Fluegen et al., 2017) modulate the characteristics of the dormant niche.

### Stem Cell or Dormant Niches

The ability of different organs to support DTC growth can be classified as dormancy-permissive or dormancy-restrictive (Bragado et al., 2012). It has been proposed that stem cell niches are specialized microenvironments that could support the survival of DTCs (Sosa et al., 2014; Ghajar, 2015; Hen and Barkan, 2020) and that they share mechanisms for cell recruitment, for example, attracting DTCs expressing CXCR4 through the secretion of G-CSF or CXCL12 in the case of prostate cancer (Shiozawa et al., 2011a). Once DTCs reach hematopoietic niches, several locally secreted factors induce DTC dormancy, such as growth arrest-specific protein 6 (GAS6) (Shiozawa et al., 2011b), bone morphogenetic protein 7 (BMP7) or BMP4 in the case of lung niches (Kobayashi et al., 2011; Gao et al., 2012), and TGFβ2 (Bragado et al., 2013).

Alternatively, dormant tumor cells acquire stem cell-like properties and they overexpress pluripotential and self-renewal genes in dormant niches (Calabrese et al., 2007; Sosa et al., 2015; Malladi et al., 2016). For example, the Notch2 pathway induces stem phenotypes resembling hematopoietic stem cells (HSCs), which mediates breast cancer cell dormancy in the endosteal niche of the bone (Capulli et al., 2019). MTOR signaling and a higher proportion of p38-MAPK relative to ERK activation is also necessary to maintain the quiescence of both DTCs and cancer stem cells (CSCs) (Aguirre-Ghiso et al., 2001; Hen and Barkan, 2020). In a model of colorectal cancer (CRC), a sub-population of quiescent cells expressing ZEB2 display stemness and mesenchymal properties, and they have been associated with chemoresistance (Francescangeli et al., 2020). FBXW7 is also expressed strongly in several populations of stem cells, sustaining lung adenocarcinoma and breast cancer dormancy by blocking entry into the cell cycle (Zhang et al., 2019). Thus, in several tumor sub-types dormant tumor cells are commonly referred to as slow-cycling CSCs that combine quiescent properties with tumor initiating and chemoresistant properties, which favor later relapse and for the formation of metastases [(De Angelis et al., 2019a, b) and references therein].

### Immune Cell-Induced DTC Dormancy

The adaptive immune system contributes directly to tumor cell dormancy in different ways (Feuerer et al., 2001; MacKie et al., 2003; Ross, 2007; Schreiber et al., 2011). It has been proposed that CD8^+^ T cells induce tumor cell dormancy and indeed, spontaneous metastases can be controlled and maintained in a dormant state by the wild-type (WT) immune system in mouse models, with no application of any anti-cancer treatment (Romero et al., 2014a). Interestingly, the recurrence of metastasis in mice depleted in T cells occurs with a much shorter latencies than in models with functional T cells (Farrar et al., 1999; Koebel et al., 2007; Eyles et al., 2010; Romero et al., 2014a; Farhood et al., 2019). A key issue in this process is whether the dormant metastases are in a quiescent state or a state of equilibrium between tumor cell proliferation and cell death, an issue not yet fully clarified. Regardless of the mechanism involved, data suggest that the interaction between MHC-I molecules on the cancer cell surface and T-cell receptors may play an important role in tumor cell dormancy (Koebel et al., 2007; Romero et al., 2014a, b). Although less significantly, CD4^+^ T cells are also involved in DTC dormancy (Romero et al., 2014a, b; Borst et al., 2018) as they induce tumor cell dormancy and cell cycle arrest through TNFR1 and IFN-γ signaling in pancreatic cancer (Muller-Hermelink et al., 2008). Additionally, CD4^+^ T cells secrete inhibitors of angiogenesis (e.g., CXCL9 and CXCL10) which can indirectly contribute to tumor dormancy by stabilizing the endothelium (Muller-Hermelink et al., 2008; Pardee et al., 2010).

The innate immune system may also be involved in tumor cell dormancy, as is the case of natural killer (NK) cells (Wu et al., 2013; Malladi et al., 2016), the cytotoxic capacity of which is mediated by perforin secretion and is very relevant in this process (Brodbeck et al., 2014). Interestingly, NK cells can also provide a variety of cytokines (e.g., CXCL10) that enhance the aforementioned ability of CD8 and CD4 lymphocytes to induce dormancy in a model of acute myeloid leukemia (AML)(Saudemont et al., 2005), however, this study shows only correlation of NK ligand expression with dormancy markers in dormant tumor masses without showing function of NK. Additionally, a recent study showed co-localization of dormancy markers (e.g., H2BK or PDGFB) with ligands of the NK group 2 member D receptor (MICA, MICB, or ULBP1 and 2) in patients with brain metastasis from lung and breast cancers, suggesting that these mechanisms may co-operate in maintaining metastatic dormancy (Fluh et al., 2020).

Other innate immune cells like macrophages are responsible for the survival of dormant breast cancer cells just after their extravasation into the lung. Aberrant expression of vascular cell adhesion molecule-1 (VCAM-1) was seen to favor the interaction of extravasated breast cancer cells with metastasis associated macrophages, an interaction that activates Akt via Ezrin, and that eventually offers metastatic cells protection against cytokine induced apoptosis (Chen et al., 2011). However, the role of this cell type in tumor dormancy remains controversial, since their polarization to a M1 or M2 phenotype seems to condition their effects on tumor cells (Yang et al., 2016).

Alternatively, dormant DTCs acquire characteristics that favor immune cell evasion and cell survival (Racila et al., 1995; Farrar et al., 1999; Muller-Hermelink et al., 2008; Teng et al., 2012; Goddard et al., 2018; Liu et al., 2018). Thus, tumor antigen expression by dormant DTCs is dampened or the expression of checkpoint inhibitors or immunosuppressive molecules induced (e.g., PD-L1, CTLA-4, CD80, CD39, and CCR4), which results in immune cell evasion (Saudemont and Quesnel, 2004; Linde et al., 2016; Malladi et al., 2016; Flores-Guzman et al., 2020). Furthermore, dormant cells decrease their MHC I levels as another means to evade T cell responses (Pantel et al., 1991; Pommier et al., 2018; Romero et al., 2018). This is a specific requirement for CD8^+^ T cell immune evasion, since MHC-I remains intact in tumor cells implanted into immunodeficient or T cell immunodepleted mice (Garcia-Lora et al., 2001; Romero et al., 2018). Interestingly, in both a pancreatic ductal adenocarcinoma mouse model and patients with liver metastasis, single DTCs do not express MHC-I or cytokeratin 19 (CK19). The downmodulation of MHC-I in dormant DTCs seems to be mediated by ER stress-dependent activation of the unfolded protein response (UPR) (Pommier et al., 2018). However, other studies suggest that MHC-I is enhanced on the surface of dormant tumor cells, generating long-term memory in CD8^+^ T cells (Perez et al., 1990; Mahnke et al., 2005; Romero et al., 2014b). Moreover, MHC-I expression on dormant cells could itself guarantee a quiescent state, since MHC-I molecules can have a direct tumor suppressor role and arrest tumor progression (Garrido et al., 2012). Together, these data suggest the possibility that both MHC-I positive and negative cells are present at dormant niches, maintained in an equilibrium that allows dormant cells to adapt to the different microenvironmental scenarios.

The presence of regulatory T cells (Tregs) in immunogenic niches might also favor the dormant niche. Interestingly, bone marrow is one of the best characterized dormant niches and it represents an important reservoir for Tregs (Zou et al., 2004). Since memory T cells could be correlated with the presence of DTCs, for example in bone metastasis of advanced breast cancer patients (Feuerer et al., 2001; Mahnke et al., 2005), it is tempting to suggest that Tregs could block the complete activation and functionality of resident memory T cells. Likewise, Treg populations increase in dormant tumors in a mouse B cell lymphoma model (BitMansour et al., 2016). Together, these data suggest that the success of the metastatic outcome depends on interactions between the immune system and DTCs, influencing the equilibrium between tumor cell proliferation and cell death (Chen et al., 2011).

### Endothelial Cell-Induced Dormancy

DTCs from various tumor types interact with the lung, bone marrow and brain vasculature at distant metastatic sites (Chambers et al., 2002; Kienast et al., 2010; Ghajar et al., 2013; Price et al., 2016; Bridgeman et al., 2017; Yadav et al., 2018). The attachment of DTCs to endothelial cells favors their survival, differentiation and the growth arrest of DTCs at dormant niches (Yadav et al., 2018). Several authors suggested that a stable endothelium may favor the survival of dormant tumors and reduce tumor growth, whereas neovascularization is associated with tumor growth (Gimbrone et al., 1972; Hawighorst et al., 2002; Folkman and Kalluri, 2004). This phenomenon could also be applied to the dormancy of micrometastases, which remain in this sleepy state when neo-angiogenesis is suppressed (Holmgren et al., 1995). Indeed, in a model of human ovarian cancer dormancy, anti-angiogenic genes are the genes most frequently affected, with enhanced TIMP3, TSP1, Ang1, and CDH1 expression as part of in dormant signature that is dampened upon tumor relapse and recurrence (Lyu et al., 2013). Thus, these data suggest that vascular niches play key roles in the fate of dormant cells. In 2013, it was demonstrated for the first time that the vascular endothelium induces tumor cell quiescence in an *in vivo* model of breast cancer dissemination (Ghajar et al., 2013), the THBS1 produced by endothelial cells promotes tumor cell dormancy. The role of THBS1 in the maintenance of tumor dormancy of breast invasive ductal carcinoma has been corroborated, pointing to tryptophan as key source for the production of THBS1 by endothelial cells (Lopes-Bastos et al., 2017). Moreover, BMP-4 signaling associated with the induction of dormancy, also augments THBS1 expression in the lung endothelium (Gao et al., 2012; Lee et al., 2014). In addition to THBS1, downregulation of VCAM1 and the lysophosphatidic acid receptor (EDG2) is also required to guarantee the dormant state of DTCs (Lu et al., 2011; Marshall et al., 2012). Interestingly, angiogenic dormancy may also contribute to tumor dormancy due to the lack of nutrients in poorly vascularized niches (Senft and Ronai, 2016; Natale and Bocci, 2018). This could control the balance between cancer cell proliferation and apoptosis. Angiostatin, a circulating inhibitor of angiogenesis, could be one of the molecules responsible for this phenomenon (O’Reilly et al., 1994; Cao et al., 1998).

Although the PVN seems to be a favorable niche for DTC dormancy, several microenvironmental cues regulate this phenomenon in specific organs. In the brain, vascular co-option of tumor cells adhered to the abluminal surface of the vasculature is strictly necessary for DTC survival (Kienast et al., 2010; Zhang et al., 2020). Moreover, astrocyte and microglial responses that promote local changes in the tumor microenvironment favor or restrict breast tumor progression (He et al., 2006; Kienast et al., 2010; Lorger and Felding-Habermann, 2010). Arrested and/or extravasated tumor cells could also activate both astrocytes and microglia in their vicinity. Astrocyte activation, identified by the up-regulation of GFAP and Nestin, leads to the expression of matrix metallopeptidase 9 (MMP-9). Since this is one of the first events in the metastatic colonization of the brain, reactive astrocyte-dependent MMP-9 secretion might create a niche that supports brain metastatic lesions (Lorger and Felding-Habermann, 2010). Alternatively, the stellate or amoeboid activated microglia cells (high F4/80 expression) could secrete multiple soluble factors that modulate both proliferative and anti-proliferative tumor responses (He et al., 2006; Lorger and Felding-Habermann, 2010). Similarly, pancreatic ductal adenocarcinoma cells use hepatic stellate cells of the sinusoidal capillaries to establish their dormancy in the liver (Lenk et al., 2017; Fabian et al., 2019). It was proposed that hepatic stellate cells induce a dormant phenotype of pancreatic ductal tumor cells through the secretion of IL-8 (Lenk et al., 2017). Indeed, hepatic stellate cells-mediated tumor quiescence is thought to also be regulated by changes in oxidative metabolism (i.e., Succinate Dehydrogenase subunit B -SDHB- expression), affecting cell growth and the stem properties of liver metastasis from pancreatic tumors (Fabian et al., 2019). In the bone, dormant cells are usually found in E-Selectin and SDF-1 rich perisinusoidal vascular areas (Price et al., 2016), which favor their entry and establishment in the bone, respectively. The close proximity of skeletal vascular networks and hepatic niches favors a microenvironment rich in ECM proteins, secreted factors like THBS1, Stem cell factor (SCF-1) or the chemokine CXCL12 that sustain tumor dormancy (Kusumbe, 2016).

In summary, the success of metastasis relies on both the microenvironment of the metastatic dormant niches and the cancer cells involved (Pencovich et al., 2013; Ghajar, 2015; Wang et al., 2015; Carlson et al., 2019). Different approaches have focused on targeting the microenvironment of metastatic dormant niches to maintain the DTCs in a quiescent state or alternatively, promoting their awakening to sensitize them to therapies (Carlson et al., 2019). Nevertheless, this scenario is very complex and unlikely to be used in the clinic at present, at least until further knowledge and a better understanding of the PVN and DTC interactions can be successfully employed to promote “eternal DTC sleepiness.”

## Influence of Extracellular Vesicles in Tumor Cell Dormancy

One of the main questions regarding the regulation of DTC dormancy concerns the mechanisms involved in the communication between stromal cells in the niche and the DTCs. EVs are thought to be important participants in intercellular communication, yet their role in the communication between DTCs and their niches is still unclear. EVs can be classified based on their origin and size. A recent classification based on size divided them into large (lEVs) and small EVs (sEVs) (Witwer and Théry, 2019), whereby microvesicles (200 nm–1 μm), apoptotic bodies (1–5 μm) and oncosomes (1–10 μm) can be considered lEVs, yet smaller vesicles like exosomes and exomeres are considered as sEVs (Witwer and Théry, 2019). EVs are a heterogeneous population of vesicles that are secreted depending on the biological context (Di Vizio et al., 2012; Zhang H. et al., 2018; Ren et al., 2019). Regardless of their origin, EVs transport proteins, lipids, and nucleic acids (both RNA and DNA) that are representative of the cell of origin (Choi et al., 2013; Raposo and Stoorvogel, 2013). Once EVs reach their target cell they can transfer their cargo horizontally, modulating physiological and pathological processes (Colombo et al., 2014). Some examples of their broad functions include their role in the cross-talk of immune cells (Thery et al., 2009), in the regulation of coagulation (Tripisciano et al., 2017) or in the formation of the pre-metastatic niche (Peinado et al., 2017). Thus, although there are only a few studies suggesting a role of exosomes in the regulation of tumor cell dormancy, there is an increase in the number of studies showing that EVs are involved in processes like vascular leakiness, extracellular remodeling and regulation of the immune system (Becker et al., 2016). Since these are processes crucial to the establishment of dormant niches, it is plausible that secreted EVs play a role regulating DTC dormancy.

### Extracellular Vesicles in the Cross-Talk Between Stem Niches and Dormant Cells

**FIGURE 1:**
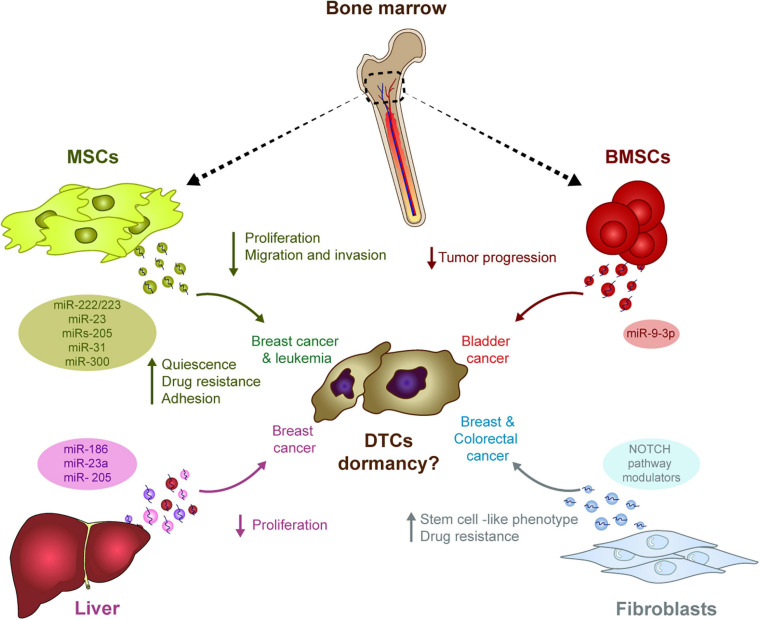
The influence of stromal-derived EVs in tumor cell dormancy. EVs generated in different stromal cells may have the ability to induce DTC dormancy in diverse tumor types. The transport of miRNA in these EVs outstand as one of the main mechanism potentially involved in this process: In the bone marrow, MSCs-derived EVs contain different miRNAs capable of modulating several pro-dormant features (e.g., quiescence, reduced proliferation, etc.) in breast cancer cells, whereas miRNAs in BMSCs-derived EVs suppress progression of bladder cancer. In the liver, several miRNAs in hepatic niche-derived EVs reduced breast cancer cell proliferation. Finally, miRNAs in EVs derived from fibroblasts induce a stem cell like phenotype and resistance to therapy in breast and colorectal cancer cells.

Homing and survival at distal sites is the most rate-limiting step in the metastatic cascade (Valastyan and Weinberg, 2011). Several studies have highlighted how stromal cell-secreted EVs influence the behavior of tumor cells in specific niches, mainly through the transfer of miRNAs (Figure 1). For example, stromal cell-secreted EVs could be differentiated from tumor-secreted EVs based on their size, their protein and miRNA content, supporting the idea of effective bi-directional cross-talk between stromal and tumor cells in the niche that sustains tumor cell colonization and dormancy (Dioufa et al., 2017). In bone marrow niches, MDA-MB-231 and T47D breast cancer tumor cells prime mesenchymal stem cells (MSCs) to release EVs containing distinct miRNAs, such as miR-222/223, which the authors propose to induce quiescence of a subset of cancer cells and confers drug resistance (Bliss et al., 2016). Of note, treatment induced only a small fraction of tumor cells into G1-G0 phase while naïve MSC-derived EVs actually induced the majority of MDA-MB-231 cancer cells into cycling. In another study, the treatment of BM2 cells (bone metastatic human breast cancer cells derived from a MDA-MB-231 parental cell line) with EVs from MSCs suppressed cell proliferation, inhibits invasion and dampens their sensitivity to docetaxel. Authors found that miR-23b was responsible of this effect by inducing dormant phenotypes through the suppression of MARKS expression (which encodes a myristoylated alanine-rich C kinase substrate) (Ono et al., 2014). In this work, however, the relevance of these findings were mainly verified *in vitro*. The *in vivo* experiments lack from verification of cell death after engraftment of metastatic breast cancer cells treated with MSC-derived exosomes. Further *in vivo* data and cell death analysis is needed to understand the relevance of these findings. Controversially, MSC EVs were only seen to suppress the metastatic potential of parental MDA-MB-231 cells but not that of metastatic MDA-231 organ tropic models (Vallabhaneni et al., 2017). In this work, authors propose that MSC EVs induced dormancy through a mechanism dependent on miRs-205 and 31, suppressing the expression of the UBE2N/Ubc13 gene that is correlated with reduced proliferation, and suppressed migration and invasion of breast cancer cells *in vitro* (Vallabhaneni et al., 2017). Nevertheless, the effect on cell death was not measured in MDA-231 cells treated with naïve exosomes from MSC and detection of remaining DTCs was not provided to analyze if DTCs were present and remained dormant in distant organs.

In addition to MDA-MB-231-derived models, treatment with hMSC EVs also reduced the proliferation and migration of MCF7 breast cancer cells, while enhancing the adhesion (Casson et al., 2018) or suppressing the progression of bladder cancer cells through ESM1 downregulation by bone marrow stem cell (BMSC)-derived exosomal miR-9-3p (Cai et al., 2019). Apart from this suggested role regulating dormancy in solid tumors, miRNAs contained in MSCs EVs have also been described to play a role in hematological malignancies. In this context, miR300 was found to contribute to the persistence of drug-resistant quiescent leukemic stem cells in chronic myelogenous leukemia. Upon miR300 upregulation in malignant cells by different sources, including EVs transference from bone marrow MSCs, leukemic cell proliferation was found to be impaired (Silvestri et al., 2020).

Hepatic niche (HepN, fresh human hepatic tissue) derived exosomes contribute to breast cancer cell homing, survival and dormancy when studied in an *ex vivo* human liver microphysiological system. In this system, MDA-231 breast cancer exosomes were used to prime the hepatic cells. Later, the primed HepN-derived EVs alter the levels of several miRNAs involved in epithelial cell differentiation (miR186, miR23a, and miR205), with a concomitant reduction of cancer cell proliferation (Dioufa et al., 2017). Exosomes from fibroblasts can also induce stem cell like features and resistance to therapy via the Notch pathway in several cancer models (e.g., breast and colorectal cancers), two intrinsic characteristics of dormant cells (Boelens et al., 2014; Hu et al., 2019).

Taking all these data together, if stromal-derived EVs influence tumor cell dormancy is a matter of current debate. We have reviewed the information in the literature and draw any conclusion would be premature at this time. Works showed that stromal cell-derived EVs may affect some characteristics of tumor cells such as proliferation, further data is needed to support their role in DTC dormacy (Figure 1). Several limitations in the interpretation must be borne in mind: (1) most of the studies analyzed cell proliferation rather than dormancy and some of the effects were only demonstrated *in vitro*; (2) the dose of exosomes and the models used (e.g., MSCs) are not always clearly defined; and (3) these studies are restricted to a limited number of cell models and immunodeficient mouse models, which limits the interpretation of the results. Further data *in vivo* and in immunocompetent models will help to understand if stroma-secreted EVs are involved in regulating tumor cell dormancy.

Similarly, while endothelial cells play a central role regulating tumor cell stemness, there is very little data suggesting that EVs secreted by endothelial cells are involved in tumor cell dormancy. In fact, endothelial cell-derived EVs have been implicated in tumor cell awakening rather than dormancy (see below, section “Similarities Between the Pre-Metastatic and the Awakening Niches”). As an exception to this statement, it was recently described that endothelial cells in the bone marrow supply miR-126 to chronic myelogenous leukemia stem cells to support quiescence, which correlates with poor prognosis. MiR-126 is highly expressed in normal HSCs and hematopoietic progenitor cells and restrains cell-cycle progression during hematopoiesis. However, in leukemic cells, miR-126 is down-regulated through a BCR-ABL-dependent mechanism. EVs from the surrounding bone marrow endothelial cells has been described as the main source to compensate the downregulation, this way allowing the reinduction of quiescence in a percentage of the leukemic cells, contributing to self-renewal, engraftment and perpetuation of the disease (Zhang B. et al., 2018). Nevertheless, since studies into tumor cell dormancy normally require an *in vivo* setting, analyzing the involvement of EVs in this process is very challenging. The development of models to properly demonstrate the influence of endothelial cell- and immune cell-derived EVs will be necessary to demonstrate their relevance, and their role *in vivo* must be compared with that of soluble and intrinsic factors.

### Extracellular Vesicles in the Regulation of Immune Dormancy

Very little is known about the role of EVs derived from immune cells in directly controlling tumor cell dormancy. There is no data in the literature linking T Cell or NK-cell derived EVs to this phenomenon. EVs derived from specific sub-populations of immune cells could have different outcomes at dormant niches. Exosomes derived from M2 macrophages reduce proliferation and limit cell cycle progression in bladder cancer models, while M1 macrophages appear to be involved in tumor cell awakening (Walker et al., 2019), as will be discussed below (see section “The Contribution of Secreted Extracellular Vesicles to Tumor Cell Awakening”). Moreover, MHC-I molecules can also be transferred between tumor and immune cells in EVs (Lynch et al., 2009; Duchler et al., 2019), potentially affecting immune responses at metastatic sites.

Interestingly, tumor-secreted EVs may contribute to immunosuppression by favoring DTC survival. Several tumor types release EVs that carry PD-L1 on their surface, mostly in the form of exosomes, and these may suppress the activity of CD8 T cells and facilitate tumor growth. Indeed, PD-L1 expression in the plasma of patients with several tumor types is correlated with a worse patient outcome and weaker immune responses (Chen et al., 2018; Theodoraki et al., 2018; Yang et al., 2018; Fan et al., 2019; Kim et al., 2019; Li C. et al., 2019; Xie et al., 2019). Therefore, tumor-secreted exosomes containing PD-L1 may potentially offer resistance to immunotherapy (Xie et al., 2019). Indeed, blockade of tumor-derived exosomal PD-L1 restores global anti-tumor immunity even in models resistant to anti-PD-L1, and this blockade suppresses tumor growth in addition to that produced by anti-PD-L1 antibodies (Poggio et al., 2019). Since dormant DTCs express less tumor antigen or induce PD-L1 expression, driving immune cell evasion (Linde et al., 2016), it is likely that DTCs can secrete PD-L1^+^ EVs and reinforce immunosuppression in niches. However, there is no data yet supporting this hypothesis in a metastatic setting.

## The Contributions of Secreted Extracellular Vesicles to Tumor Cell Awakening

A particular challenge when considering metastasis is to understand which signals are involved in the outgrowth of quiescent DTCs (Braun et al., 2000). Multiple signals are involved in the reactivation of silent DTCs through a process known as re-awakening (Aguirre-Ghiso, 2018). This metastatic cell re-awakening leads to the generation of secondary lesions, which in many cases constitute the cause of death of cancer patients (Chaffer and Weinberg, 2011). Although this condition has been largely documented by clinicians, the molecular mechanisms underlying this process and specially, the contribution of EVs to these events, are still to be defined. Successful metastasis requires a supportive microenvironment in which the DTCs can proliferate, otherwise these cells will remain quiescent in these niches (Luzzi et al., 1998; Shibue et al., 2012; Lambert et al., 2017).

### Potential Involvement of EVs in DTC Awakening

**FIGURE 2:**
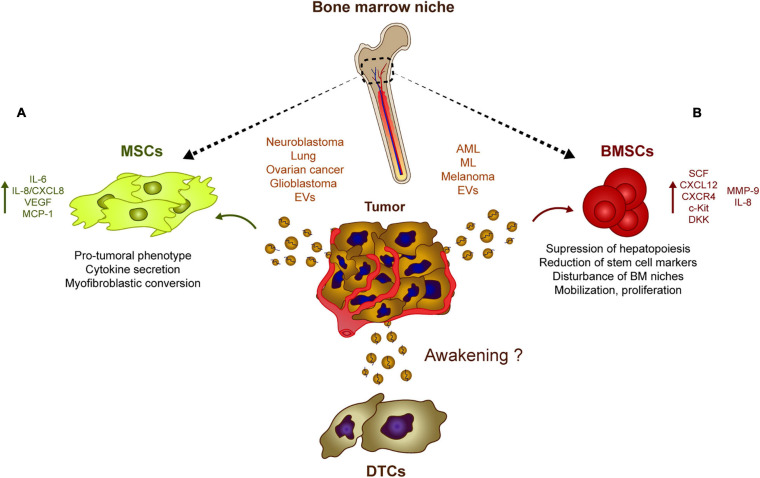
Tumor-derived EVs influence stromal populations. Rather than inducing DTC dormancy, tumor-derived EVs could contribute to their re-awakening through different mechanisms. **(A)** Neuroblastoma, glioblastoma, lung, and ovarian cancer generate EVs capable of inducing a pro-tumoral phenotype in MSCs which usually involves a myofibroblastic conversion. These modified MSCs eventually upregulate the secretion of pro-tumoral cytokines and chemokines such as IL-8, IL6, and VEGF. **(B)** Similarly, melanoma or leukemia cells release EVs that are captured by BMSCs which induces the secretion of IL8, CXCL12 among other soluble factors. This tumor EV-mediated release of cytokines and chemokines has an effect on the bone marrow physiology suppressing hematopoiesis and disturbing several features of the bone marrow progenitors, which favor the growth of tumor cells at dormant niches.

As mentioned previously, stromal cell-derived EVs may be involved in DTC dormancy *in vitro*, whereas tumor-derived EVs may contribute to tumor progression and could be involved in tumor cell awakening (Figure 2). It was recently shown that neuroblastoma-derived EVs (Nakata et al., 2017) and lung cancer-derived EVs (Li et al., 2016) are captured by bone marrow-derived MSCs *in vitro*, inducing the secretion of pro-tumoral cytokines and chemokines like interleukin-6 (IL-6), IL-8/CXCL8, vascular endothelial cell growth factor (VEGF) and monocyte-chemotactic protein-1 (MCP-1) (Li et al., 2016; Nakata et al., 2017). Similarly, sEVs derived from ovarian cancer spheroids (with a cancer stem cell phenotype) modify the activity of MSCs and induce the secretion of IL-6, IL-8, and VEGF-A. Interestingly, exposure to cisplatin alters the cargo of sEVs released by ovarian cancer tumor cells, inducing a pro-tumorigenic activity of MSCs *in vitro* (Vera et al., 2019). Elsewhere, exosomes from ovarian cancer cell lines were seen to induce a myofibroblastic phenotype and activity in MSCs by activating different intracellular signaling pathways depending on the model used (Cho et al., 2011). Overall, it seems that tumor-derived EVs co-opt MSCs and re-program them to produce a pro-tumorigenic activity. The growth of these re-programmed MSCs is enhanced directly, as is the horizontal growth of fibroblasts, endothelial cells and immune cells in the tumor microenvironment, indirectly promoting the pro-tumor activity of MSCs (Whiteside, 2018). The secretion of IL-6, IL-8, and VEGF, and the activation of pro-inflammatory pathways seem to be the two canonical mechanisms by which MSCs respond to tumor cell-derived exosomes. Interestingly, glioblastoma-derived EVs also induce an increase in the secretion of VEGF and IL-6, as well as an increase in the phagocytotic capacity of macrophages, reinforcing their tumor-supportive phenotypes (de Vrij et al., 2015). Analyzing the relevance and importance of EVs relative to other molecules *in vivo* will be crucial to understand if tumor-derived EVs could act as “awakeners” (Figure 2) as opposed to the potential role of MSC-derived EVs in “sleepy” niches (Figure 1).

Similarly, there is growing evidence that leukemic cells can modulate the EVs in their host bone marrow microenvironment to survive and expand. AML-derived EVs suppress normal hematopoiesis by inhibiting protein synthesis and they induce long-term hematopoietic stem cell quiescence through the internalization of miR-1246 (Abdelhamed et al., 2019) or by inducing the expression of DKK1, a suppressor of normal hematopoiesis (Kumar et al., 2018). Exosomes also regulates hematopoietic stem and progenitor cells indirectly by decreasing the SCF and CXCL12 in bone marrow-derived MSCs, or by reducing CXCR4 and c-Kit expression, triggering the ensuing suppression of hematopoietic transcription factors like c-Myb, Cebp-β, and Hoxa-9 (Huan et al., 2015; Hornick et al., 2016). In addition, amphiregulin enriched exosomes from chronic myelogenous leukemia augment the adhesion and proliferative advantage of tumor cells within the hematopoietic niche by mediating the expression of MMP-9 and IL-8 (Corrado et al., 2016). Moreover, melanoma-derived EVs are involved in the mobilization of bone marrow progenitor cells and in pre-metastatic niche formation by enhancing c-Met signaling in hematopoietic progenitors, disturbing the natural physiology of bone marrow niches (Peinado et al., 2012). These studies suggest that tumor-derived EVs affect the normal physiology of hematopoietic and mesenchymal stem cells, preceding tumor cell invasion, and helping to promote tumor cell proliferation and survival within the niches (Figure 2).

### Similarities Between the Pre-metastatic and the Awakening Niches

It has been proposed that the primary tumor is involved in the preparation of secondary organs for the arrival and growth of tumor cells, known as the pre-metastatic niche (PMN) (Peinado et al., 2017). During the last decade, exosomes have been proposed as key players in this process (Kahlert and Kalluri, 2013; Becker et al., 2016; Lobb et al., 2017; Steinbichler et al., 2017). Interestingly, many of the modifications influenced by secreted EVs could also affect DTC awakening, such as: (1) Increasing in metastatic behavior, tumor cell survival and angiogenesis; (2) promoting ECM remodeling; (3) and favoring the recruitment of bone marrow-derived cells and local inflammation described during the generation of PMNs (Peinado et al., 2017). The similarities between both processes, raises interesting questions, such as could similar mechanisms be involved in the generation of PMNs and in DTC re-awakening? Could EVs be involved in DTC re-awakening together with other factors?

#### The Role of EVs in Metastasis, Tumor Cell Survival and Angiogenesis

**FIGURE 3:**
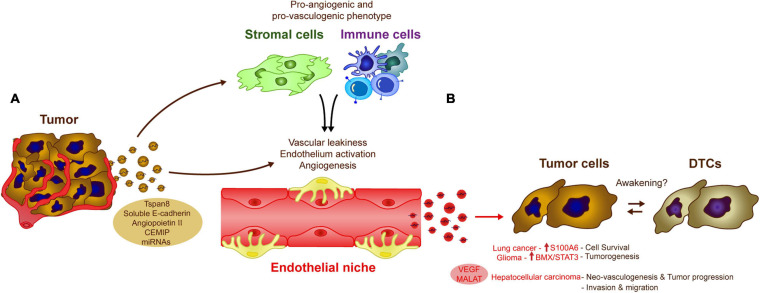
Influence of endothelial cell-derived EVs in tumor cells. The crosstalk between tumor and endothelial cells via EVs modulate several biological processes that could influence DTC awakening. **(A)** Tumor-derived exosomes induce vascular leakiness, endothelium activation and angiogenesis, key processes in metastasis development, directly by transferring different molecules and miRNAs to the endothelial cells or indirectly by affecting other cell populations in the microenvironment as stromal or immune cells. **(B)** The altered endothelium is also capable of generating EVs, which induce pro-metastatic features (e.g., cell survival, neoangiogenesis, invasion) in a variety of cancer types (as Lung cancer, glioma, or hepatocellular carcinoma), these signals may be similar to those involved in tumor DTC awakening.

There is evidence that endothelial cell-derived EVs influence tumor cells and for example, human brain microvascular endothelial-derived exosomes are thought to favor lung cancer tumor cell survival and resistance to apoptosis by increasing the levels of S100A16 (Xu et al., 2019). In a model of glioma, CD9-enriched EVs derived from endothelial cells also enhance glioma stem cell tumorigenesis by activating the BMX/STAT3 axis (Li D. et al., 2019). YAP1 depletion or inhibition in vascular endothelial cells increases the release of exosomes that contain the long non-coding RNA MALAT1 into the tumor microenvironment (Li et al., 2020). Exosomal transfer of MALAT1 to hepatic cells favors hepatic cell invasion and migration due to the activation of extracellular signal-regulated kinase 1/2 (ERK1/2) signaling (Li et al., 2020). Interestingly, HUVEC release VEGF-enriched exosomes that may combat anti-angiogenic treatments, a phenomenon that favors tumor neo-vasculogenesis and tumor progression in hepatocellular carcinomas (Zeng et al., 2019). These data raise an interesting question, since endothelial cells are involved in tumor cell dormancy, could their secreted EVs take part in processes related to tumor cell awakening? One potential explanation is that while the physical interactions between stable endothelial and tumor cells are crucial in early tumor cell homing and to induce DTC dormancy, as the disease evolves, sprouting of endothelial cells could promote the secretion of EVs involved in DTC awakening in the endothelial niches, together with other factors (Figure 3; Risson et al., 2020).

Tumor-derived EVs also contribute to tumor angiogenesis and the disruption of the endothelial vascular barrier. Cell-to-cell communication between cancer cells and endothelial cells via EVs, is one of the most relevant mechanisms promoting vascular leakiness, angiogenesis and vascular remodeling reported in the literature (Kikuchi et al., 2019). Tumor-derived EVs, carry proteins like Tspan8 (Nazarenko et al., 2010), soluble E-cadherin (Tang et al., 2018), or Angiopoietin II (Xie et al., 2020) that are directly involved in the activation of the endothelium, or indirectly through the induction of pro-vasculogenic phenotypes in stromal cells (e.g., fibroblasts) (Cho et al., 2012; Chowdhury et al., 2015) or pro-angiogenic immune cells (Peinado et al., 2012). Breast cancer-derived exosomes expressing the cell migration-inducing and hyaluronan-binding protein (CEMIP) induce endothelial cell branching and inflammation in brain endothelial and microglial cells, upregulating the pro-inflammatory cytokines Ptgs2, Tnf, and Ccl/Cxcl that have been previously implicated in brain vascular remodeling and metastasis (Rodrigues et al., 2019). Similarly, the shedding of RNAs by EVs appears to be one of the most important mechanisms involved in angiogenesis associated with a wide variety of tumors (Skog et al., 2008; Umezu et al., 2014; Conigliaro et al., 2015; Zeng et al., 2018; Deng et al., 2020). Together, these studies suggest that tumor-derived EVs are important regulators of angiogenesis, a process that may be triggered after tumor cell awakening in endothelial cell niches (Figure 3).

#### Role of EVs on ECM Remodeling

Another hallmark of PMN formation that may be also relevant to tumor cell awakening is ECM remodeling (Peinado et al., 2017). Fibronectin deposition directly correlates with reactivation of proliferative processes in dormant cells (Aguirre-Ghiso et al., 2001). Therefore, tumor-derived exosomes may contribute to DTC awakening by altering the ECM components and provoking abnormal deposition of molecules like fibronectin (Mu et al., 2013; Costa-Silva et al., 2015; Sung et al., 2015), collagen, laminin (Mu et al., 2013), annexins, and integrins (Hoshino et al., 2015; Keerthikumar et al., 2015). These modifications are crucial to recruit inflammatory cells, which eventually contribute to ECM degradation by secreting proteinases into metastatic niches, particularly MMPs (Rucci et al., 2011). Indeed, tumor-derived exosomes induce MMP activity, aiding ECM remodeling (Shimoda, 2019; Deep et al., 2020). Proteomic analysis has shown that these proteases may be directly transported by EVs from both the tumor and the microenvironment, playing a direct role in ECM remodeling (Taraboletti et al., 2002; Sanderson et al., 2019; Shimoda, 2019). Therefore, it is likely that these activities could be involved in the contribution of local niches to DTC awakening.

#### The Role of EVs in the Recruitment of Bone Marrow-Derived Cells and Local Inflammation

**FIGURE 4:**
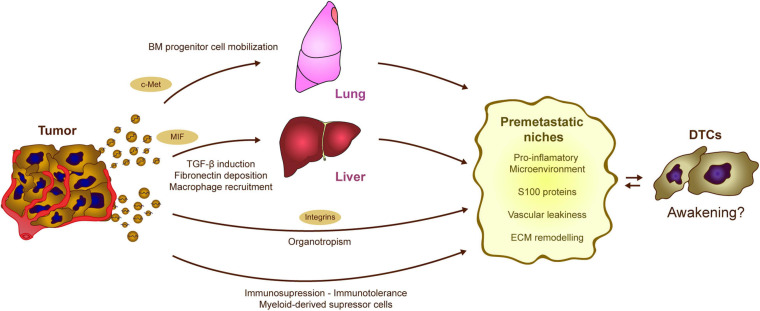
Potential contribution of tumor-EVs to DTC awakening. The processes regulated by tumor derived EVs in formation of PMNs could be involved in DTC awakening. Tumor exosomes induce the mobilization of bone marrow precursors, which eventually contribute to the generation of pro-inflammatory and immunosuppressive niches. Similarly, alterations in the stromal cells of the liver result in the generation of a fibrotic and pro-tumorigenic microenvironment. Moreover, both PMN and sleepy niches are organotropic processes that occur at specific locations along the body depending on the tumor type. Interestingly, the expression of different integrins on the EVs surface seems to mediate metastatic organotropism. Overall, tumor derived exosomes trigger different processes as the induction of pro-inflammatory microenvironments, vascular modification, and EMC remodeling which result into the generation of optimal pre-metastatic niches. These processes may affect the equilibrium between dormancy and awakening in the DTCs.

Another important mechanism for the generation of PMNs relies on the effect of tumor secreted exosomes on the immune system (Figure 4). Abnormal mobilization of immune cells, as well as the ability to switch them toward a pro-metastatic phenotype, contribute to the progression of different tumor types. The ability of tumor-derived EVs to modify homeostasis of the immune system and induce pro-inflammatory signals in different tissues facilitates both tumor and immune cell recruitment. An initial analysis of melanoma-derived exosomes demonstrated that they are involved in the mobilization of bone marrow-progenitor cells to PMNs, reinforcing metastatic behavior (Peinado et al., 2012). Melanoma-derived exosomes reprogram bone marrow progenitors toward a pro-vasculogenic phenotype, which involves the oncogene c-Met, and they induce pro-inflammatory genes in the lungs to favor the generation of PMNs (Peinado et al., 2012). Later, it was found that the generation of PMNs in the liver is promoted by Kupffer cells taking up pancreatic cancer-derived exosomes carrying MIF (Costa-Silva et al., 2015). Exosome uptake triggers the secretion of TGFβ, which in turn activates fibronectin production and deposition by hepatic stellate cells. This fibrotic microenvironment attracts bone marrow derived F4/80^+^ macrophages, which eventually enhance metastasis (Costa-Silva et al., 2015). It was later found that pro-inflammatory niches could be induced by reinforcing the expression of different S100 proteins in specific organs (Hoshino et al., 2015). Indeed, exosomes expressing integrins α6β4 and α6β1 preferentially home to the lung, while exosomes expressing integrin αvβ5 home to the liver, reinforcing PMN formation, as well as S100 protein induction and metastasis in those organs (Hoshino et al., 2015). *In vivo* treatment with exosomes derived from the EO771 model breast cancer cells also modifies the proportions of immune cells in the lungs. These EVs increase the frequency of macrophages and myeloid derived suppressor cells (MDSCs), while diminishing the numbers of CD8 T cells and NK cells, skewing the microenvironment toward an immunosuppressive state and promoting metastasis (Wen et al., 2016). In addition, recent evidence reinforces the importance of specific molecular cargos. In contrast to highly metastatic tumors, EVs derived from poorly aggressive melanomas appear to recruit completely different populations of immune cells to the PMNs, promoting immunosurveillance based on the expansion of Ly6C^low^ patrolling monocytes and NK cell recruitment, a phenomenon that results in cancer cell clearance and that thereby averts metastasis (Plebanek et al., 2017).

Exosomes exert different effects on immune cell behavior that could awaken DTCs. Besides the tumor exosomes ability to mobilize bone marrow derived cells (Peinado et al., 2012; Huan et al., 2015), they may also stimulate dormant DTCs in the bone marrow itself (Vora et al., 1994). Apart from the already mentioned mechanism of immune recruitment upon ECM remodeling (Erler et al., 2009; Yan et al., 2010; Costa-Silva et al., 2015), exosomes can directly stimulate the stroma to actively secrete chemotactic signals, as happens in alveolar cells via Toll-like receptor 3 activation (Liu et al., 2016) or in MSCs through ERK1/2 and AKT induction (Nakata et al., 2017). Once within the PMN, tumor exosomes have the ability to modulate the immune response toward immunotolerance via different mechanisms, as extensively reviewed elsewhere (Whiteside, 2016a; Olejarz et al., 2020). To do so, tumor exosomes can block the differentiation of myeloid and lymphoid progenitors, promote pro-tumorigenic macrophage polarization, or contribute to the expansion of immunosuppressive populations like Treg lymphocytes or MDSCs (Whiteside, 2016a). The combination of immune cell recruitment and the induction of an immunosuppressive microenvironment together with ECM remodeling and vascular leakiness are key factors in PMN formation, strongly modulated by tumor EVs. All these alterations may eventually compromise immunosurveillance and disrupt tumor cell dormancy, preceding metastatic outgrowth (Figure 4; Muller et al., 1998; Baxevanis and Perez, 2015). However, the specific implications in DTC re-awakening still remain to be elucidated.

## Exosomes as Biomarkers of DTC Dormancy and Awakening: The Future of Liquid Biopsies

Exosomes are considered potential biomarkers in oncological diseases (Whiteside, 2016a; Huang and Deng, 2019), and there is evidence that analyzing the exosomes in liquid biopsies could be a promising technique to detect mutations or to monitor residual disease in different biofluids (Whiteside, 2016b; Castellanos-Rizaldos et al., 2018). Indeed, a recent report showed that EVs carry specific biomarkers of cancers that can be identified with high sensitivity and efficiency in cancer patients (Hoshino et al., 2020). Hence, defining molecular signatures in liquid biopsies from patients may help identify cancer patients with a higher risk of relapse. Taking into account the non-invasive and easy ways that liquid biopsies can be obtained (Gold et al., 2015), this concept could also be applied to monitor the risk of late relapses in disease-free cancer patients. Although tumor dormancy presents challenges for clinical diagnosis and a great opportunity for therapeutic approaches, there are few studies that have focused on new methods to detect dormancy early. Defining and detecting dormant-related signatures, or pro-inflammatory and pro-awakening signatures, should be explored as clinic criteria to manage patients prior to disease reappearance (Console et al., 2019), complementing those currently existing (Sansone et al., 2017).

Liquid biopsy is defined by the analysis of biological material of tumor origin that extravasate to body fluids. Most common liquid biopsy are focused on the use of circulating tumor cells (CTCs) or circulating tumor-derived factors, in particular, circulating tumor DNA (ctDNA) (Alix-Panabières and Pantel, 2016; Husain and Velculescu, 2017; Perakis and Speicher, 2017; Pantel and Alix-Panabières, 2019). The quantification of circulating DNA molecules or CTCs showed *per se* prognostic value in many cancers (Haber and Velculescu, 2014). Another advantage of using CTCs and ctDNA resides mainly in the possibility of testing specific mutations, methylation profiles, and other DNA patterns (ctDNA and CTCs) and alternatively, proteins and the possibility of generating patient-derived xenografts (PDX) from the most aggressive cells in the tumor that putatively could initiate metastatic outgrowth (CTCs) (Alix-Panabières and Pantel, 2016; Husain and Velculescu, 2017; Perakis and Speicher, 2017; Pantel and Alix-Panabières, 2019). Furthermore, the development of high sensitivity and specificity techniques enabled the identification of minimal residual disease (MRD) in cancer patient’s follow-up blood samples (Pantel and Alix-Panabières, 2019). Complementary to these biomarkers, EVs emerged in the last years as powerful biomarkers to provide information about the tumor and the systemic changes occurring during the disease.

Due to their heterogeneous content (protein, nucleic acids, lipids, metabolites, etc.), their ubiquitous production by body cells and detection in most biological fluids, circulating EVs could be useful for specific or multiplatform analyses to provide an accurate evaluation of cancer disease at early time points, during progression, therapy and post-treatment facilitating the detection of MRD and relapse anticipation (LeBleu and Kalluri, 2020).

Each technique has its own pros and cons. For example, CTCs have been mainly used to understand the biology of early metastatic spread and resistance to established therapies, they require higher amount of plasma and specific equipment for isolation and detection (Pantel and Alix-Panabières, 2019). ctDNA analysis in plasma of cancer patients allows the identification of genomic alterations, monitoring of treatment responses, unraveling therapeutic resistance, and potentially detecting disease progression before clinical confirmation is obtained (Pantel and Alix-Panabières, 2019). In recent years a huge effort has been made to compare ctDNA and EV-DNA in order to provide a better understanding of their applications. An important limitation of EV-DNA studies is that, while ctDNA analysis is already standardized in the clinic with multiple platforms, there is a lack of standardized isolation methods of EV-DNA analysis that require clinical validation (Théry et al., 2006; Royo et al., 2020). While EV-DNA is more stable and less fragmented (Kalra et al., 2013) than ctDNA (Mouliere et al., 2011; Cheng et al., 2016; Lazaro-Ibanez et al., 2019), to date, most liquid biopsy have been performed in ctDNA (Cristiano et al., 2019; Poore et al., 2020; Sprang et al., 2020). Finally, the main advantage of EVs-based liquid biopsy is the possibility to concentrate the circulating material by specific protocols and perform multiplexing analyses of DNA with other EV cargo such as RNA that can provide a highly accurate information about the disease and will facilitate the use of personalized medicine approaches (LeBleu and Kalluri, 2020; García-Silva et al., 2021).

In the particular case of dormancy, distinguish circulating dormant cells could represent an arduous task due to the limited number of these cell populations. Similarly, defining the EV signatures related to “dormant” and “awakened” tumor cells is far from their use in the clinic. Since miRNAs in circulating EVs have diagnostic and/or prognostic potential for many cancer types, this may be a potential way to identify novel biomarkers. In exosomes isolated from plasma/serum of cancer patients, miR17-92a and miR-19a are correlated with increased colon cancer recurrence or a worse prognosis (Matsumura et al., 2015). Exosomal circulating miR141 and miR375 have been associated with metastatic prostate cancer and treatment outcome (Zedan et al., 2020), while high levels of exosomal miR-21 can predict esophageal cancer recurrence and distal metastasis (Liao et al., 2016), and this phenomenon is associated with cisplatin resistance in ovarian cancer (Pink et al., 2015). Alternatively, the down-regulation of miR-125b is correlated with metastatic melanomas (Alegre et al., 2014). Since stromal cells may fulfill a decisive role in supporting tumor dormancy through the release of miRNA containing EVs, it is tempting to speculate that analyzing the expression of miRNAs in circulating EVs could serve as a biomarker for “dormant stages” (Ono et al., 2014; Bliss et al., 2016; Vallabhaneni et al., 2017), nevertheless this is just a hypothetical scenario at this time. Since the detection of DTCs and their EV signatures is proving difficult due to their relative low abundance in circulation, defining dormant signatures based on stromal EVs could represent a more effective approach due to the concentration of material obtained after EV isolation protocols.

Similarly, defining an EV signature related to DTC awakening could potentially be of interest. While CTCs have been used successfully in the last years to detect early metastatic cell awakening, there are several limitations in sensitivity that must be solved in the case of EVs to play a relevant role in this scenario (Pantel and Alix-Panabières, 2019). Due to the strong connection between awakening and PMN formation, these combined studies could also include certain exosomal markers of PMN, some of which have already been correlated with poor prognosis, such as c-MET in the case of lung metastasis in melanoma patients (Peinado et al., 2012) or MIF in the case of liver metastasis of pancreatic cancer (Costa-Silva et al., 2015).

One of the main advantages of using liquid biopsies to detect awaked DTCs is that can be identified in pre-symptomatic patients after surgery, opening the possibility of using them as an early marker and screening tool of MRD. In addition, non-invasive liquid biopsies confer the advantage and the opportunity to follow patients during and after treatment, providing an accurate and real time read-out of tumor development with a minimal risk to patients. CTCs and ctDNA have demonstrated their use for MRD detection in plasma (Pantel and Alix-Panabières, 2019). In addition to these techniques, we recently examined the use of DNA mutations in EVs and ctDNA isolated from the drainage implanted post-lymphadenectomy to detect MRD in melanoma patients (Garcia-Silva et al., 2019). These approaches suggest that combination of several fractions (e.g., ctDNA and EV-DNA/RNA), together with the use of novel biofluids anatomically closer to tumor sites (seroma from drainage post-lymphadenectomy) and specific isolation methods (that allows the concentration of material), increases the sensitivity of detection.

Defining the signals released by the awakened DTCs could define novel biomarkers that help to identify MRD. However, liquid biopsies to determine dormancy have several limitations. First, since DTCs remain at low numbers within niches, the representation of their EVs in the bloodstream could be confused with EVs released from normal tissues. Second, it is not clear whether all tumor sub-clones at different metastatic locations will secrete and distribute EVs equally into the bloodstream, or if this might be influenced by other factors, such as the extravasation properties of the tissues they have colonized. Third, there is heterogeneity in the dormant niches within tissues in terms of how their surrounding cell microenvironment promotes the quiescence or reawakening of DTCs. Fourth, works shown in this section come from patients that had already reactivation of the residual disease and disruption of dormancy. In order to detect signals of tumor reawakening by liquid biopsy a strong effort should be made to design new protocols and collect samples (e.g., plasma) in a routinely basis (e.g., yearly) after surgery to follow up disease reactivation.

Thus, it is important to expand the dormant EV signatures to stromal EVs rather than just limiting these to tumor-derived dormant EVs. Moreover, optimized protocols for EV isolation and cargo analysis are required, including the use of novel biofluids or combined sources [e.g., CTCs, ctDNA with EV-DNA (Garcia-Silva et al., 2019)].

## Concluding Remarks

Despite the potential of exosomes to regulate a wide variety of biological and pathological processes, there is still little information regarding their involvement in tumor cell dormancy and awakening. As revised here, there are only a few studies that support this idea, the main limitation when interpreting these studies is that they are normally performed *in vitro*. Thus, it is necessary to overcome this limitation and demonstrate their relevance *in vivo*. In addition, metastasis in human patients has traditionally been addressed once the secondary lesion has been detected, meaning that dormancy had already been disrupted. Hence, human data concerning DTC awakening is very scarce (Linde et al., 2016) and the relevance of EVs remains to be defined.

Importantly, more data is needed to frame these ideas in a clinical context. Are the stromal EVs relevant in patients carrying premalignant lesions? or in patients with already invasive primary tumors? The majority of papers shown favor more the later since they mainly used evolved tumor cells (e.g., MDA-231 cells). Another main question to be solved is whether dormant niches may be influenced by naïve stroma-derived EVs or alternatively if dormant niches may be influenced by stroma-derived EVs after being primed by exosomes from primary tumors or primed by the first arriving DTCs.

Regarding the involvement of endothelial cell-derived EVs in DTC awakening, works do not clarify if these interactions are happening during early or late DTC dissemination. The clinical context is also hard to depict for these interactions. How it is possible that EVs may be relevant years after tumor resection? Is pre-metastatic niche once created able to lasts for years, even decades? Can tumor cells be influenced by endothelial cell-derived EVs in this setting? or Can DTC secrete enough EVs to trigger their own awakening? Are external factors involved (e.g., stress, neutrophil extracellular traps (NET) formation)? While both awakening and PMN formation involve tumor cells and their microenvironment, the molecular mechanisms involved in DTC awakening and the involvement of EVs have yet to be formerly demonstrated.

Another crucial issue that remains to be demonstrated is the potential to detect tumor cell awakening-derived EVs in liquid biopsies. The identification of biomarkers to detect patients with an active disease due to DTC awakening could have a major impact in the clinic. While this is plausible, several limitations must be overcome, and as such, it will be necessary to identify biomarkers secreted by “awakened” DTC that can be assessed and to confirm that they can be detected in liquid biopsies. Similarly, their correlation with disease outcome must be defined.

Thus, it is still early to determine the true potential of liquid biopsies to detect tumor cell awakening or their derived EVs. Similarly, defining the relevance of EVs derived both from tumor and stromal cells in tumor cell awakening and therapy resistance would help establish their relevance in the clinical setting.

## Author Contributions

HP developed the idea and wrote the manuscript. AH-B wrote the manuscript and designed the figures. LN wrote the manuscript. All authors contributed to the article and approved the submitted version.

## Conflict of Interest

The authors declare that the research was conducted in the absence of any commercial or financial relationships that could be construed as a potential conflict of interest.

## Glossary

**Abbreviation:** Description

**Akt:** AKT; serine/threonine Kinase

**AML:** Acute myeloid leukemia

**Ang1**: Angiopoietin 1

**BCR-ABL**: Breakpoint cluster region/Abelson protooncogene translocation (a.k.a Philadelphia chromosome)

**BM2**: Breast cancer cell line BM2 (derived from the breast cancer cell line MDA-MB-231)

**BMP4**: Bone morphogenetic protein 4

**BMP7**: Bone morphogenetic protein 7

**BMSC:** Bone marrow stem cell

**BMX:** BMX non-receptor tyrosine kinase

**CAFs:** Cancer-associated fibroblasts

**Ccl:** C-C Motif Chemokine Ligand

**CCR4**: C-C motif chemokine ligand 4

**CD4^+^ T cells**: Helper T lymphocytes

**CD8^+^ T cells**: Cytotoxic T lymphocytes

**CD9**: Cluster of differentiation 9

**CD39**: Cluster of differentiation 39

**CD80**: Cluster of differentiation 80

**CDH1**: Cadherin 1

**Cebp-β**: CCAAT enhancer binding protein beta

**CEMIP:** Cell migration inducing hyaluronidase 1

**CK19**: Cytokeratin 19

**c-Kit**: KIT proto-oncogene; receptor tyrosine kinase

**c-Met**: MET proto-oncogene; receptor tyrosine kinase

**c-Myb**: MYB proto-oncogene; transcription factor

**CRC:** Colorectal cancer

**CSCs:** Cancer stem cells

**CTCs:** Circulating tumor cells

**ctDNA:** Circulating tumor DNA

**CTLA-4**: Cytotoxic T-lymphocyte associated protein 4

**Cxcl:** C-X-C motif chemokine ligand

**CXCR4**: C-X-C motif chemokine receptor 4

**CXCL8**: C-X-C motif chemokine ligand 8

**CXCL9**: C-X-C motif chemokine ligand 9

**CXCL10**: C-X-C motif chemokine ligand 10

**CXCL12**: C-X-C motif chemokine receptor 12

**DKK1**: Dickkopf WNT signaling pathway inhibitor 1

**DTCs:** Disseminated tumor cells

**ECM:** Extracellular matrix

**EDG2**: Lysophosphatidic acid receptor

**EO771**: Breast cancer cell line EO771

**ER:** Endoplasmic reticulum

**ERK:** Extracellular signal-regulated kinase

**ERK1/2**: Extracellular signal-regulated kinase 1/2

**EVs:** Extracellular vesicles

**F4/80**: A.k.a ADRE1 Adhesion G Protein-Coupled Receptor E1

**FBXW7**: F-Box and WD repeat domain containing 7

**GAS6**: Growth arrest-specific protein 6

**G-CSF**: Granulocyte colony-stimulating factor

**GFAP:** Glial fibrillary acidic protein

**H2BK**: Histone cluster 1 H2B family member K

**HepN:** Hepatic niche

**Hoxa-9**: Homeobox A9

**HSCs:** Hematopoietic stem cells

**HUVEC:** Human umbilical vein endothelial cells

**IFN-γ**: Interferon gamma

**IL-6**: Interleukin-6

**IL-8**: Interleukin-8

**lEVs:** Large extracellular vesicles

**Ly6C**: Lymphocyte antigen 6 complex

**M1**: Macrophage phenotype 1

**M2**: Macrophage phenotype 2

**MALAT1**: Metastasis associated lung adenocarcinoma transcript 1

**MARKS:** Myristoylated alanine-rich C kinase substrate

**MCP-1:** Monocyte-chemotactic protein-1

**MDA-231**: Breast cancer cell line MDA-231

**MDA-MB-231**: Breast cancer cell line MDA-MB-231

**MDSCs:** Myeloid derived suppressor cells

**MHC-I**: Major histocompatibility complex-I

**MICA:** MHC class I polypeptide-related sequence A

**MICB:** MHC class I polypeptide-related sequence B

**MIF:** Macrophage migration inhibitory factor

**miRNAs:** MicroRNA

**MMP-9**: Matrix metallopeptidase 9

**MRD:** Minimal residual disease

**MSCs:** Mesenchymal stem cells

**mtDNA:** Mitochondrial DNA

**mTOR:** Mechanistic target of rapamycin kinase

**NK:** Natural killer

**Notch2**: Notch Receptor 2

**p38-MAPK**: p38 mitogen-activated protein kinase

**PDGFB:** Platelet derived growth factor subunit B

**PD-L1**: Programmed death-ligand 1

**PMN:** Pre-metastatic niche

**Ptgs2**: Prostaglandin-endoperoxide synthase 2

**PVN:** Perivascular niche

**S100**: S100 calcium binding proteins

**S100A16**: S100 calcium binding protein A16

**SCF-1**: Stem cell factor

**SDF-1**: Stromal cell–derived factor 1

**SDHB:** Succinate dehydrogenase subunit B

**sEVs:** Small extracellular vesicles

**STAT3**: Signal transducer and activator oft transcription 3

**T47D**: Breast cancer cell line T47D

**TGFβ:** Transforming growth factor-β

**THBS1**: Thrombospondin-1

**TIMP3**: Tissue Inhibitor of metalloproteinases 3

**Tnf:** Tumor necrosis factor

**TNFR1**: Tumor necrosis factor receptor superfamily member 1A

**Tregs:** Regulatory T lymphocytes

**Tspan8**: Tetraspanin 8

**UBE2N/Ubc13**: Ubiquitin conjugating enzyme E2 N

**ULBP1**: UL16 binding protein 1

**UPR:** Unfolded protein response

**VCAM-1**: Vascular cell adhesion molecule-1

**VEGF:** Vascular endothelial cell growth factor

**VEGF-A:** Vascular endothelial growth factor A

**WT:** Wild-type

**YAP1**: Yes1 associated transcriptional regulator

**ZEB2**: Zinc finger E-box binding homeobox 2

**α6β1**: Integrin subunit alpha 6/beta 1

**α6β4**: Integrin subunit alpha 6/beta 4

**αvβ5**: Integrin subunit alpha v/beta 5.
